# Assessment of malaria control consultation and service posts in Yunnan, P. R. China

**DOI:** 10.1186/s40249-016-0185-y

**Published:** 2016-10-04

**Authors:** Xu-Can Zeng, Xiao-Dong Sun, Jian-Xiong Li, Meng-Ni Chen, Dao-Wei Deng, Cang-Lin Zhang, Zu-Rui Lin, Zi-You Zhou, Yao-Wu Zhou, Ya-Ming Yang, Sheng Zhou

**Affiliations:** 1 Yunnan Institute of Parasitic Disease, 6 Xiyuan Road, Pu’er, 665000 Yunnan China; 2 Key Laboratory of Surveillance and Early-warning on Infectious Disease, Division of Infectious Diseases, Chinese Center for Disease Control and Prevention, 155 Changbai Road, Changping District, Beijing, 102206 People’s Republic of China

**Keywords:** Malaria, Border areas, Mobile and migrant population, Examination stations, Yunnan, Myanmar, China

## Abstract

**Background:**

This paper seeks to assess the function of malaria control consultation and service posts (MCCSPs) that are located on the border areas of Yunnan province, P.R. China, as a strategy for eliminating malaria among the mobile and migrant population in these areas.

**Methods:**

A retrospective descriptive analytical study was conducted. Blood smear examinations conducted at all MCCSPs in Yunnan from 2008 to 2014 were analysed. A cross-sectional survey was conducted in 2014 to understand how the MCCSPs function and to elucidate the quality of the blood smear examinations that they conduct.

**Results:**

Out of the surveyed MCCSPs, 66 % (39/59), 22 % (13/59), and 12 % (7/59) were attached to local township hospitals, village health clinics, and the county centre for disease control and prevention or private clinics, respectively. More than 64 % (38/59) of the posts’ staff were part-time workers from township hospitals and village health facilities. Less than 31 % (18/59) of the posts’ staff were full-time workers. A total of 35 positive malaria cases were reported from seven MCCSPs in 2014. Four MCCSPs were unable to perform their functions due to under staffing in 2014. There was a small fluctuation in blood smear examinations from January 2008 to June 2009, with two peaks during the period from July 2009 to October 2010. The number of blood smear examinations has been increasing since 2011. The yearly mean number of blood smear examinations in each post increased from 44 per month in 2011 to 109 per month in 2014, and the number of positive malaria cases detected by blood smear examinations has declined (*χ*^2^ = 90.67, *P* = 0.000). The percentage of people from Yingjiang county getting blood smear examinations increased between 2008 and 2014, while percentages of the mobile population including Myanmar people, people from other provinces, and people from other Yunnan counties getting blood smear examinations decreased.

**Conclusion:**

MCCSPs face challenges in the phase of malaria elimination in Yunnan, China. New case detection strategies should be designed for MCCSPs taking into account the current trends of migration.

**Electronic supplementary material:**

The online version of this article (doi:10.1186/s40249-016-0185-y) contains supplementary material, which is available to authorized users.

## Multilingual abstract

Please see Additional file 1 for translation of the abstract into the five official working languages of the United Nations.

## Background

Malaria is an important public health issue in China. The malaria elimination program commenced in 2010, and from that year reported malaria cases have decreased dramatically from 7 389 to 2 921 in 2014. However, the number of imported malaria cases rose from 72 % of all cases in 2010 to 98 % of all cases in 2014.

An imported malaria case is defined as a person with a malaria infection that can be traced to an origin in a malaria-endemic area outside of China and that was reported within 1 month of returning from an endemic area. About 20–30 % of imported malaria infections are from countries of the Greater Mekong Subregion (GMS) [1]. In 2014, 533 malaria cases were reported in Yunnan. Imported cases accounted for 91.2 % (*n* = 486) of these, of which 79.6 % (387) were caused by the *Plasmodium vivax* parasite. The majority of these *P. vivax* imported malaria infections (88.89 %; *n* = 344) came from Myanmar [2].

Epidemiological data provided by malaria programs show a drastic decline in malaria deaths and confirmed malaria positive cases over the last 10 years in the GMS [3]. This has prompted the World Health Organization (WHO) to develop a malaria elimination strategy for the subregion for 2015–2030 [4].

Yunnan province has the highest burden of malaria in China, and because it shares its border with a number of countries and due to the movement of populations across these borders, this poses a challenge for malaria control efforts [5]. The province shares a 4061 km borderline with Myanmar, Laos, and Vietnam, and 19 counties administered by six prefectures share a 1997 km border line with Myanmar’s Kachin and Shan states. Border crossings are made easy in Yunnan thanks to the long borderline and mountainous areas, and it is estimated that several millions of people belonging to the mobile and migrant population (MMP) cross the border each year [6, 7]. This means that MMPs pose a great challenge for malaria elimination in China [8, 9].

With financial support from the Global Fund to Fight AIDS, Tuberculosis and Malaria (GFATM), 48 Malaria Control Consultation and Service Posts (MCCSPs) were established in October 2007 in 12 border counties in five prefectures in Yunnan. The objective of the posts is to reduce the malaria burden on mobile Chinese workers by providing comprehensive malaria prevention and care services for the target Chinese mobile population. These posts primarily aim to early detect and promptly treat malaria cases, administer health education, and provide long-lasting insecticidal nets and malaria prophylaxis for MMPs.

In order to better control malaria among MMPs, since 2012, a total of 66 MCCSPs were established in 19 border counties in six prefectures in Yunnan, with financial support from the Chinese government. Although since then management of the MCCSPs has been the same, as it was during the period of support from the GFATM, the malaria situation in the area has changed. Currently, imported malaria cases are of utmost concern and early malaria case detection by the MCCSPs in Yunnan is one effective measure to help eliminate malaria in China. Reviewing program effectiveness and assessing the performance of these posts is essential to not only ensure that more effective services are provided for MMPs but also that the risk of reintroducing malaria is reduced by better targeting imported cases in the phase of malaria elimination in China.

## Methods

### Study areas

**Fig. 1:**
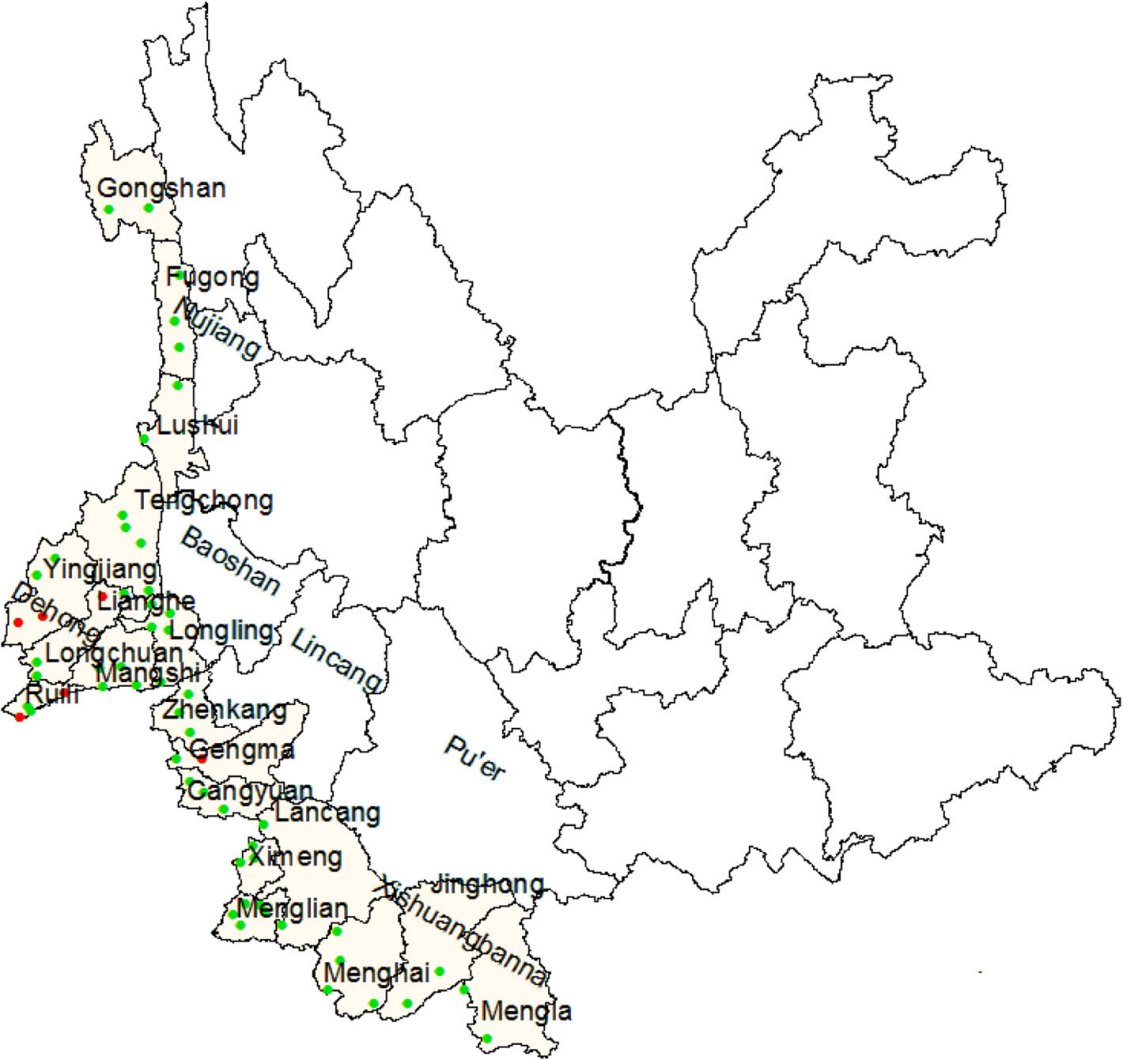
Malaria Control Consultation and Service Posts location. Legend:  MCCSP that reported positive malaria cases in 2014.  MCCSP that reported negative malaria cases in 2014

We conducted a retrospective descriptive analytical study to understand how the 66 MCCSPs in 19 counties that border Yunnan and Myanmar are functioning and how effective their programming is. These counties are located in the Baoshan, Dehong, Nujiang, Lincang, Pu’er, and Xishuangbanna prefectures (see Fig. 1).

### Data collection

We collected annual data on malaria cases in Yunnan from the Yunnan Institute of Parasitic Diseases (YIPD) for the period of 2008–2014. The collected data was on blood smear examinations, and their results, conducted by the MCCSPs, and the length of workers’ employment at the posts. The aim was to compare the data from the period of the GFATM funding phase and the Chinese government funding phase.

A cross-sectional survey was conducted among 59 out of the 66 MCCSPs from January 6 to February 10, 2015. Seven MCCSPs could not be reached due to poor transportation. According to the microscopy quality assurance system in China, all positive blood smear slides have to be rechecked by microscopists at the county and provincial levels. Meanwhile, 10 and 1 % negative slides should be rechecked by microscopists at the county and provincial level, respectively. Ten blood smear slides from each MCCSP were collected to assess the quality of the blood smear examinations at the 59 MCCSPs. A qualified microscopist from YIPD read the slides and ranked them either as ‘good’, ‘not bad’, and ‘poor’, according to the quality of smear making, dyeing, and the cleanliness of the blood film.

### Statistical analysis

Data were analysed using Stata software, version 10.0 (Stata Corp, College Station, TX, USA). The chi square test for trend was used to determine trends of positive blood smears among the years. The chi square test (*χ*^2^) was also used to compare post workers’ length of employment among the MCCSPs. Results were considered significant at the 5 % critical level (*P* < 0.05).

## Results

### General information about the MCCSPs

As of 2014, there was a total of 66 MCCSPs in 19 counties in six prefectures in Yunnan province (see Fig. 1). We surveyed 59 of these as seven were unreachable.

About 66 % (39/59) of the MCCSPs were attached to local township hospitals, 22 % (13/59) were attached to local village health clinics, and almost 12 % (7/59) were attached to other health facilities, such as the county Center for Disease Control and Prevention (CDC), private hospitals, and so on.

More than 64 % (38/59) of the posts’ staff were hospital workers and village doctors. Nearly 31 % (18/59) were newly recruited full-time employees. Two people were CDC staff and one person worked at a MCCSP for his retirement. (see Table 1).

**Table 1:** Characteristics of MCCSP

Category	No.	%
Location		
Township hospital	39	66
Village health clinic	13	22
Other health facilities	7	12
Professionals		
Part time staff from hospital staff	25	42
Part time staff from village doctors	13	22
Recruitment of full time staff	18	31
Part time staff from CDC	2	3
Full time staff from retired person	1	2
blood smear quality		
Good	10	17
Not bad	22	37
poor	27	46
Working period (*n* = 219)		
≤ 1 year	50	23
≤ 2 years	63	29
≤ 3 years	42	19
≥ 4 years	64	29

### Performance of the MCCSPs

In 2014, seven MCCSPs reported 35 positive malaria cases out of 77 215 fever cases (see Fig. 1). Four posts were unable to perform their functions due to under staffing.

During the period of 2008–2014, there was a seasonal variation in the reported malaria cases in the 19 border counties, with the peak period of malaria transmission decreasing each year.

**Fig. 2:**
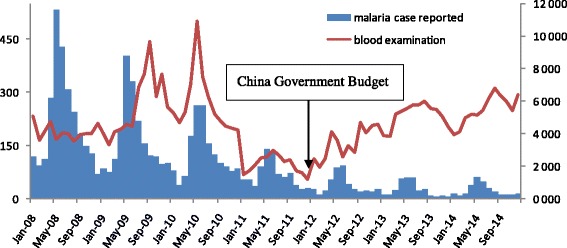
Distribution of malaria case reported in the 19 counties and blood smear examination in 66 malaria control consultation and service posts in 2008–2014

The number of blood smear examinations among fever patients varied. The lowest number of blood smears was reported in 2011 (see Fig. 2). The yearly mean number of blood smear examinations increased from 44 per month in 2011 to 109 per month in 2014, and the number of positive malaria cases detected by blood smear examinations declined (*χ*^2^ = 90.67, *P* = 0.000) (see Table 2).

**Table 2:** Summary of malaria control consultation and service posts in 2008–2014

	2008	2009	2010	2011	2012	2013	2014
No. of blood smear examed in the 19 counties^a^	181 500	189 028	186 739	134 564	148 152	149 583	151 607
Malaria Case reported^b^	2 653	2 015	1521	803	447	303	281
No. of blood smear examinated by MCCSP^c^	49 437	68 544	70 178	25 191	41 061	61 838	77 215
No. of positive result^d^	–	–	–	–	99	79	35
Yearly Mean blood smears (/month/post)*^c^	78	119	122	44	52	78	109

^a^There are totally 48malaria control consultation and service posts in 2008–20011 and 66 MCCSP in 2012–2014

^b^Malaria case reported in the 19 counties

^c^Blood smear examination includes a few RDTs

^d^Positive slides reported by 66 MCCSP. ^*^ The number of positive result in 2008–2010 is not available due to different case definition

In terms of the quality of the blood smear examinations, 17 % (10/59) of MCCSPs conducted blood smears of good quality, 37 % (22/59) conducted blood smears of not bad quality, and 46 % (27/59) conducted blood smears of poor quality. During the period of 2008–2014, the average length of employment (working days) of all 219 employees recorded was 1 864, 1 075, 1 180, 1 022, 815, and 992 at the MCCSPs in Baoshan, Dehong, Lincang, Nujiang, Pu’er, and Xishuangbanna, respectively. The average working days of employees were significantly different among the MCCSPs in the six prefectures (*χ*^2^ = 108.91, *P* = 0.000).

**Fig. 3:**
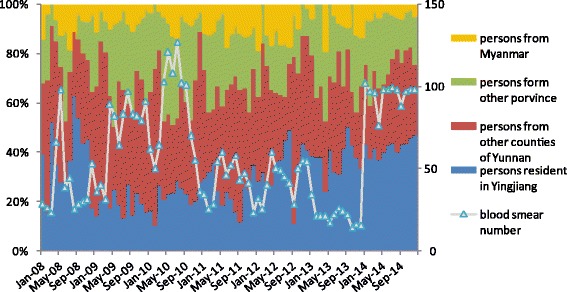
Comparison of the percentage origin residence among blood smear examination in Nab in 2008–2014

The percentage of people from Yingjiang county getting blood smear examinations increased over the study period, while percentages of the mobile population including Myanmar people, people from other provinces, and people from other Yunnan counties getting blood smear examinations decreased (see Fig. 3). Similar numbers of blood smear examinations were conducted at the Naban post in the different months of 2014, while a seasonal peak was observed from May to October between 2008 and 2013 in Yingjiang. Local residents of Yingjiang accounted for about 40 % in 2014, while the average figure of local residents of Yingjiang was 30 % during the period of 2008–2014 (see Fig. 3).

## Discussion

Yunnan has made great achievements in malaria control with help from the GFATM. Malaria incidence dropped from 21.79/100 000 in 2000 to 0.95/100 000 in 2014. Yunnan reported a total of 3 484 malaria cases over the period of 2011–2014, with imported malaria cases making up 85 % (2 958/3 484) of these. *Plasmodium vivax* was the predominant species, accounting for 72 % (2 500/3 484) of the cases and *P. falciparum* accounted for 20 % of the cases in this period. Malaria cases reported in Yunnan border areas are mainly transmitted by MMPs. Individual case investigation showed that 90 % (1 786/1 984) of imported malaria cases with apparent infection sources were infected in Myanmar [10].

According to statistics from the WHO, about 6 000 people are infected with malaria every year in Myanmar SAR. More than half of the confirmed malaria cases and deaths recorded in the GMS are from Myanmar. *P. falciparum* is a major species in Myanmar, accounting for 40.94 % of the total malaria cases, which is quite a bit higher than that in Yunnan. More and more imported cases of *P. falciparum* malaria are being reported from Myanmar. For example, in 2005 the reported number of *P. falciparum* malaria cases in Yunnan accounted for 84 % of the total *P. falciparum* cases in China (3 497/4 146), with 69 % (2 413/3 497) of these being imported from neighbouring countries along the Yunnan border [11]. A higher number of imported *P. falciparum* cases will increase the risk of death from malaria.

MCCSPs are important for servicing MMPs in Yunnan. Although MCCSPs in Yunnan undertook activities successfully as indicated by the figures of the blood smear examinations conducted in 2014, they also faced challenges. According to our survey, a few posts were not functioning, which shows management issues at some of the MCCSPs. Performance-based management should be introduced in the MCCSPs and posts with poor performance should be replaced or suspended. Secondly, almost half of the posts conducted malaria case detection poorly. Poor quality blood smear examinations make microscopic diagnosis difficult. Those MCCSPs conducting good quality blood smear examinations were mainly located in Dehong and Baoshan. The reason may be because Kachin state, which is opposite Dehong and Baoshan, has the highest malaria incidence in Myanmar. This means that the staff at these posts probably received sufficient on-the-job training for conducting blood smear examinations. As a result, those post have a better blood smear quality during routine work. Posts that conducted poor quality blood smear examinations were mainly located in Lincang, Pu’er, and Xishuangbanna. The reason may be due to the Shan state having a lower malaria incidence rate compared to Kachin. Moreover, a high turnover of staff and unqualified staff may also account for the poor quality of blood smear examinations conducted at the MCCSPs in Lincang, Pu’er, and Xishuangbanna. On the one hand, these results suggested that more training and supervision should be provided from higher levels, such as from prefecture and provincial facilities. On the other, this survey showed that staff changes frequently at MCCSPs and that recruitment of staff seems to be a major concern. Lacking incentive may be another reason for this trend, considering that most staff members are from public health facilities and only work at the MCCSPs part time.

The GFATM ceased its funding for China in 2011, which is the reason why that year saw the lowest number of blood smear examinations in the study period. Malaria elimination activities have been budgeted for by the central government since 2012 and some changes have occurred alongside this change in financial support. For Chinese MMPs engaged in logging and planting banana activities in Myanmar, new strategies are needed [12, 13]. A previous study indicated that there is a considerable number of malaria cases in China-Myanmar border areas that remain undiagnosed or misdiagnosed by microscopy, especially when it comes to low-level and/or complex co-infection cases [14]. It is of utmost urgency to develop accurate rapid diagnostic tests (RDTs) because they are a major solution for improving efficiency for diagnosing malaria, especially at posts that conduct poor quality blood smear examinations. In addition, fixed-schedule mobile clinics should be set up on border crossings for screening and treatment of malaria in hotspots after the adoption of RDTs [15, 16]. Reaching out to highly mobile migrants with health messaging and malaria diagnosis and treatment services is imperative in order to contain the spread of artemisinin resistance in *P. falciparum* malaria [17]. Moreover, blood smear examinations should target MMPs. It is unusual that there were similar numbers of blood smear examinations conducted at the Naban post in the different months in 2014. Although it is not advisable to make a conclusion based on one post, several posts from the other five counties also recorded similar numbers of blood smear examinations in the different months in 2014. Unfortunately, more detailed data were not available from the other five counties. Increasing the number of blood smear examinations at local households without considering the risk factors may not be helpful for early case detection in the phase of malaria elimination in China.

Although the National Malaria Elimination Program has yielded remarkable achievements in 2014, the number of imported malaria cases has increased significantly. The border areas of Yunnan are still key areas for malaria elimination in China. At the same time, domestically-mobile cases should be further managed [2]. The GMS has become one of the fastest growing regions in the world, with improved regional transport networks, such as roads and railways, facilitating increased cross-border trade and travel. As a result, a massive increase in population movements across borders in the GMS will happen [18]. Strategies for early malaria case detection for MMPs will need to consider changes in migration patterns during the phase of malaria elimination in China.

## Conclusion

Due to increased border crossings between Myanmar and Yunnan, MCCSPs face new challenges in the phase of malaria elimination in China. Increasing the number of blood smear examinations at these posts plays a limited role in early case detection among MMPs in Yunnan border areas. At the posts that conduct poor quality blood smear examinations, RDTs need to be utilized and/or strengthened. New case detection strategies should be designed for MCCSPs taking into account the current trends of migration.

## Additional file

Additional file 1: Multilingual abstract in the five official working languages of the United Nations. (PDF 602 kb)

## Abbreviations

CDC: Center for disease control and prevention
GFATM: Global fund to fight AIDS, tuberculosis and malaria
GMS: Greater mekong subregion
MCCSP: Malaria control consultation and service post
MMP: Mobile and migrant population
RDT: Rapid diagnostic test
WHO: World Health Organization
YIPD: Yunnan institute of parasitic diseases
