# Supplementary material for: Assessment of malaria control consultation and service posts in Yunnan, P. R. China
Source: Infect Dis Poverty. 2016 Oct 4;5:102. doi: 10.1186/s40249-016-0185-y (PMC5048452; doi:10.1186/s40249-016-0185-y)
Supplement: Additional file 1: — Multilingual abstract in the five official working languages of the United Nations. (PDF 602 kb) [file 40249_2016_185_MOESM1_ESM.pdf]

Translation of the abstract into the five official working languages of the United Nations

تقييم الوظائف الاستشارية والخدمية لمكافحة الملاريا في يونان بجمهورية الصين الشعبية

شو كان تسنغ، وشياو دونغ سون، وجيان زيونغ لى، ومنغ ني تشن، وداو وي دينغ، وتسانغ لين تشانغ، وزو روي لين، وتسى يو زو، وتشو ياو وو زو، ويا مينغ يانغ وتشينغ زو

تلخيص

معلومات عامة: وتسعى هذه الدراسة إلى تقييم وظيفة استشارة القضاء على الملاريا ومواقع الخدمة التي تقع على المناطق الحدودية لمقاطعة يونان، في جمهورية الصين الشعبية، باعتبارها استراتيجية للقضاء على الملاريا بين السكان المتنقلين والمهاجرين في هذه المناطق.

الطرق: قد أجريت دراسة تحليلية وصفية بأثر رجعي. فقد جرى تحليل فحوصات مسحة الدم التي أجريت على الوظائف الاستشارية والخدمية لمكافحة الملاريا في يونان ما بين 2008-2014. وأجري مسح قطاعي في عام 2014 لفهم الكيفية التي تعمل بها الوظائف الاستشارية والخدمية لمكافحة الملاريا وتوضيح نوعية فحوصات مسحة الدم التي يقومون بإجرائها.

النتائج: ومن نتائج استبيان الوظائف الاستشارية والخدمية لمكافحة الملاريا التي شملتها الدراسة، ارتبطت نسبة 66٪ (59/39) و 22٪ (59/13)، و 12٪ (59/7) بالمستشفيات المحلية بالبلدات والعيادات الصحية في القرى، ومركز مقاطعة لمكافحة الأمراض والوقاية منها أو العيادات الخاصة، على التوالي. وكان أكثر من 64٪ (59/38) من العاملين بالوظائف من "العاملين بدوام جزئي في مستشفيات البلدة والمرافق الصحية بالقرية. وشغل أقل من 31٪ (59/18) من العاملين بالوظائف من "العاملين بدوام كامل. كما تم الإبلاغ عن ما مجموعه 35 حالة من الملاريا الإيجابية لدى سبعة من العاملين في الوظائف الاستشارية والخدمية لمكافحة الملاريا في عام 2014. ولم يتمكن أربعة من العاملين فيالوظائف الاستشارية والخدمية لمكافحة الملاريا من أداء مهامهم بسبب نقص الموظفين في عام 2014. وكان هناك تذبذب طفيف في فحوصات مسحة الدم خلال الفترة من يناير 2008 إلى يونيو 2009، مع وجود ذروتين خلال الفترة من يوليو 2009 إلى أكتوبر 2010. وكان عدد فحوصات مسحة الدم قد تزايد منذ عام 2011. وارتفع متوسط عدد فحوصات مسحة الدم سنويًا في كل وظيفة ارتفعت من 44 في الشهر في عام 2011 إلى 109 في الشهر في عام 2014، كما انخفض عدد اكتشاف حالات الملاريا إيجابية الكشف عنها بواسطة فحص مسحة الدم (X^2=90.67, *P*=0.000). وقد ارتفعت النسبة المئوية للأشخاص من محافظة يينغجيانغ الحاصلين على فحوصات مسحة الدم بين عامي 2008 و 2014، في حين انخفضت نسبة السكان المتنقلين بما في ذلك شعب ميانمار، والمواطنين من محافظات أخرى، والمواطنين من مقاطعات يونان الأخرى الحاصلين على فحوص مسحة الدم.

الاستنتاج: تواجه الوظائف الاستشارية والخدمية لمكافحة الملاريا تحديات في مرحلة القضاء على الملاريا في يونان بالصين. إستراتيجيات رصد الحالة الجديدة التي ينبغي وضعها من أجل الوظائف الاستشارية والخدمية لمكافحة الملاريا مع الوضع في الحسبان الاتجاهات الحالية للهجرة.

Translated from English version into Arabic by Fathia Sobhi, through

TRANSLATORS
WITHOUT BORDERS

中国云南疟疾咨询服务站评估

曾旭灿，孙晓东，李建雄，陈梦妮，邓道伟，张苍林，林祖锐，周子悠，周耀武，杨亚明，周升

摘要

引言: 本文对作为消除疟疾策略的中国云南省边境的疟疾咨询服务站进行评估
方法：对 2008 年至 2014 年云南省的所有疟疾咨询服务站进行回顾描述性分析研究。
结果：在被调查的疟疾咨询服务站中，66% (39 个/59 个)设在当地乡镇卫生院, 22% (13 个/59 个) 设在村卫生室, 12% (7 个/59 个)设在县疾控中心或私人诊所。超过 64% (38 个/59 个)的人员来自乡镇卫生院和村卫生室的兼职人员。低于 31% (18/59)的站点工作人员为专职工作者。2014 年 35 例疟疾病例来自 7 个疟疾咨询服务站的报告，但 2014 年 4 个疟疾咨询服务站没能开展工作。从 2008 年一月至 2009 年 6 月血涂片镜检有小幅的波动，在 2009 年 7 月至 2010 年间有 2 个高峰。从 2011 年起血涂片镜检数开始增长，在 2014 年疟疾咨询服务站的血涂片镜检平均达到 109 张/月，同时镜检阳性片下降(X^2=90.67, P=0.000)。盈江县接受血检的人口比例从 2008 年至 2014 年是增加的，但是流动人口（包括缅甸人，外省人和云南其他县的人）接受血检的人比例却是下降的。
结论:中国云南疟疾咨询服务站在消除疟疾阶段面临着挑战。应该把目前的人员流动趋势考虑在内，设计新的病例检测策略。

Translated from English version into Chinese by Xu-Can Zeng and Sheng Zhou

Évaluation des postes de consultation de lutte contre le paludisme et de service au Yunnan, en Chine

Xu-Can Zeng, Xiao-Dong Sun, Jian-Xiong Li, Meng-Ni Chen, Dao-Wei Deng, Cang-Lin Zhang, Zu-Rui Lin, Zi-You Zhou, Yao-Wu Zhou, Ya-Ming Yang, et Sheng Zhou

RÉSUMÉ

Contexte : Cet article vise à évaluer la fonction des postes de consultation de lutte contre le paludisme et de service (PCLPS) situés dans les zones frontalières de la province du Yunnan, en République Populaire de Chine, en tant que stratégie d'éradication du paludisme parmi la population mobile et migrante de ces régions.
Méthodes : Une étude analytique descriptive rétrospective a été menée. Les examens par frottis de sang effectués de 2008 à 2014 dans tous les PCLPS du Yunnan ont été analysés. Une enquête transversale a été réalisée en 2014 pour savoir comment fonctionnent les PCLPS et déterminer la qualité des examens par frottis de sang qu'ils effectuent.
Résultats : Parmi les PCLPS étudiés, 66 % (39/59), 22 % (13/59) et 12 % (7/59) étaient attachés à des hôpitaux locaux du canton, à des cliniques de village, au centre de contrôle et de prévention des maladies du comté, ou à des cliniques privées, respectivement. Plus de 64 % (38/59) des employés des postes travaillaient à temps partiel dans des hôpitaux du canton et des établissements de santé de village. Moins de 31 % (18/59) des membres du personnel des postes travaillaient à temps plein. Au total, 35 cas positifs pour le paludisme ont été signalés dans sept PCLPS en 2014. Quatre PCLPS étaient incapables d'exercer leurs fonctions en raison d'une insuffisance d'effectifs en 2014. Il y avait une légère fluctuation du nombre d'examens par frottis de sang de janvier 2008 à juin 2009, avec deux pics au cours de la période entre juillet 2009 et octobre 2010. Le nombre d'examens par frottis de sang a augmenté depuis 2011. Le nombre annuel moyen d'examens par frottis de sang dans chaque poste est passé de 44 par mois en 2011 à 109 par mois en 2014, et le nombre de cas

positifs pour le paludisme détectés par les examens par frottis de sang a diminué (X^2=90,67, p=0,000). Le pourcentage de personnes du comté de Yingjiang ayant eu des examens par frottis de sang a augmenté entre 2008 et 2014, alors que les pourcentages de la population mobile ayant eu des examens par frottis de sang ont diminué, en comprenant les personnes originaires du Myanmar, d'autres provinces et d'autres comtés du Yunnan.
Conclusion : Les PCLPS font face à des défis dans la phase d'éradication du paludisme au Yunnan, en Chine. De nouvelles stratégies de détection de cas devraient être conçues pour les PCLPS, en tenant compte des tendances migratoires actuelles.

Translated from English version into French by Jacek Sierakowski, through

TRANSLATORS
WITHOUT BORDERS

Оценка результатов консультационных и обслуживающих пунктов по борьбе с малярией в провинции Юньнань (КНР)

Сюй-Кан Цзэн, Сяо-Дун Сунь, Цзянь-Сюн Ли, Мэн-Ни Чжэнь, Дао-Вэй Дэн, Цан-Лин Чжан, Цзу-Жуй Лин, Цзы-Ю Чжоу, Яо-У Чжоу, Я-Мин Ян и Шэн Чжоу

КРАТКОЕ ИЗЛОЖЕНИЕ

История вопроса: В данном исследовании рассматриваются результаты работы консультационных и обслуживающих пунктов по борьбе с малярией (КОПБМ), расположенных на приграничных территориях провинции Юньнань в КНР, используемых в целях ликвидации малярии среди перемещающейся и мигрирующей части населения.
Методы: Было проведено ретроспективное описательное аналитическое исследование. Были проанализированы мазки крови, забранные во всех КОПБМ в провинции Юньнань в период с 2008 по 2014 гг. В 2014 г. было проведено перекрестное исследование с целью анализа результатов работы КОПБМ и выявления количества анализов мазков крови, которое они проводят.
Результаты: Из всех рассмотренных КОПБМ 66% (39 из 59), 22% (13 из 59) и 12% (7 из 59) были прикреплены к местным городским больницам, /деревенским клиникам, окружным санитарно-эпидемиологическим центрам и частным клиникам, соответственно. В более 64% (38 из 59) пунктов сотрудники являлись работающими на неполную ставку работниками городских больниц или деревенских клиник. В менее 31% (18 из 59) пунктов сотрудники работали на полную ставку. 35 положительных случаев малярии были зарегистрированы семью пунктами в 2014 г. Четыре пункта не справлялись с работой по причине нехватки персонала в 2014 г. С января 2008 г. по июнь 2009 г. наблюдалось небольшое колебание в заборе мазков крови, а с июля 2009 г. по октябрь 2010 г. наблюдалось два периода повышения. Ежегодное среднее количество анализов мазков крови в каждом пункте повысилось с 44 в месяц в 2011 г. до 109 в месяц в 2014 г., а количество положительных анализов мазков на малярию снизилось (X^2=90,67, P=0.000). Процент людей из района Инцзян, сдающих мазки

крови, повысился в период с 2008 по 2014 гг., а процент мигрирующего населения, включая мьянманцев, жителей других провинций и других округов провинции Юньнань, сдающих мазки крови, понизился.
Заключение: Пункты КОПБМ сталкиваются с трудностями на стадии ликвидации малярии в провинции Юньнань в Китае. Необходимо разработать новые стратегии для КОПБМ, принимая во внимание текущие миграционные тенденции.

Translated from English version into Russian by Elena McDonnell, through

TRANSLATORS WITHOUT BORDERS

Evaluación del control de la malaria y de puestos de servicios de consulta en Yunnan, República Popular China

Xu-Can Zeng, Xiao-Dong Sun, Jian-Xiong Li, Meng-Ni Chen, Dao-Wei Deng, Cang-Lin Zhang, Zu-Rui Lin, Zi-You Zhou, Yao-Wu Zhou, Ya-Ming Yang y Sheng Zhou

SÍNTESIS

Historia: En este trabajo se pretende evaluar la función de las consultas de control de la malaria y puestos de servicio (en inglés MCCSP) que se encuentran en las zonas fronterizas de la provincia de Yunnan, República Popular de China, como una estrategia para eliminar la malaria entre la población móvil y migrante en estas áreas.
Métodos: Se llevó a cabo un estudio analítico descriptivo retrospectivo. Se analizaron los exámenes de manchas de sangre realizados a todos los MCCSP en Yunnan entre 2008 y 2014. Se llevó a cabo una encuesta transversal en 2014 para entender cómo funcionan los MCCSP y para verificar la calidad de los exámenes de manchas de sangre que se realizan.
Resultados: De los MCCSP encuestados, el 66% (39/59), 22% (13/59) y 12% (7/59) formaban parte de hospitales locales, clínicas de salud municipales, localidades y centros municipales para el control de enfermedades y prevención o clínicas privadas, respectivamente. Más del 64% (38/59) del personal eran trabajadores a tiempo parcial en hospitales municipales y centros de salud ubicados en pueblos. Menos del 31% (18/59) de personal eran trabajadores a tiempo completo. Un total de 35 casos positivos de malaria se registraron en siete MCCSP durante el 2014. Cuatro MCCSP fueron incapaces de realizar sus funciones debido a la falta de personal durante el 2014. Se detectó una pequeña fluctuación en los exámenes de manchas de sangre entre enero de 2008 junio de 2009, con dos picos durante el período entre julio de 2009 octubre de 2010. El número de exámenes manchas de sangre ha ido en aumento desde 2011. El número anual medio de los exámenes de manchas de sangre en cada puesto ha aumentado de 44 por mes en 2011 a 109 por mes en 2014 y el número de casos positivos de malaria detectados por los exámenes de machas de sangre ha disminuido (X^2= 90.67, P= 0.000). El porcentaje de personas del condado de Yingjiang que se sometieron a exámenes de manchas de sangre aumentaron entre 2008 y 2014, mientras que los porcentajes de la población móvil, incluyendo gente de Myanmar, personas de otras provincias y

personas de otros condados de Yunnan que se sometieron a exámenes de manchas de sangre disminuyeron.

Conclusión: Los MCCSP enfrentan problemas en la fase de eliminación de la malaria en Yunnan, China. Deben de ser diseñadas nuevas estrategias de detección de casos para los MCCSP, teniendo en cuenta las tendencias actuales de migración.

Translated from English version into Spanish by Francisco Gonzalez, through

TRANSLATORS
WITHOUT BORDERS
